# A review of the botany, phytochemistry, pharmacology, synthetic biology and comprehensive utilization of *Silybum marianum*

**DOI:** 10.3389/fphar.2024.1417655

**Published:** 2024-07-11

**Authors:** Xiaozhuang Zhang, Meiqi Liu, Zhen Wang, Panpan Wang, Lingyang Kong, Jianhao Wu, Wei Wu, Lengleng Ma, Shan Jiang, Weichao Ren, Likun Du, Wei Ma, Xiubo Liu

**Affiliations:** ^1^ College of Pharmacy, Heilongjiang University of Chinese Medicine, Harbin, China; ^2^ First Affiliated Hospital, Heilongjiang University of Chinese Medicine, Harbin, China; ^3^ College of Jiamusi, Heilongjiang University of Chinese Medicine, Jiamusi, China

**Keywords:** *Silybum marianum*, phytochemistry, pharmacology, synthetic biology, comprehensive utilization

## Abstract

*Silybum marianum* (L.) Gaertn, a herbaceous plant with a long history in traditional medicine for the treatment of hepatobiliary diseases, particularly in Europe, which has attracted attention for its remarkable therapeutic effect. This review systematically summarizes the research progress in the botany, phytochemistry, pharmacology, comprehensive utilization and synthetic biology of *S*. *marianum*. Up to now, more than 20 types of flavonolignan components have been isolated from *S*. *marianum*. In addition, the rearch on fatty acids and triterpenoids is also constantly improving. Among them, silybin is the most active compound in flavonolignans components. Its pharmacological effects *in vivo* and *in vitro* include anti-inflammatory, antioxidant, anti-tumour, hypoglycaemic, neuroprotective and immunoregulatory properties. The use of coniferyl alcohol and taxifolin as substrates to produce silybin and isosilybin under the action of enzyme catalysis is the commonly used biosynthetic pathway of silymarin, which provides support for a comprehensive analysis of the synthetic pathway of silymarin. In addition to medicinal use, the extracts of plants also have broad application prospects in the production of food, healthcare products, cosmetics and other aspects. In addition, the chemical composition, pharmacological mechanism and synthetic biology of *S. marianum* need to be further studied, which is very important for its clinical efficacy and resource development.

## 1 Introduction

**FIGURE 1:**
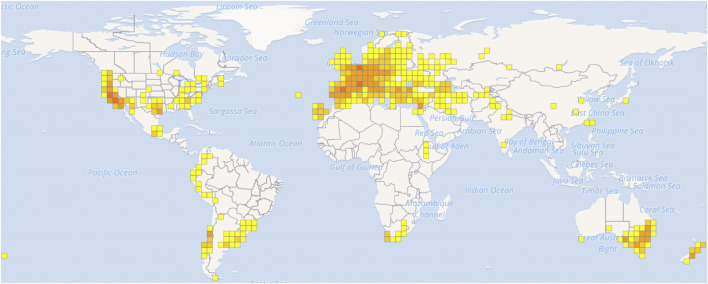
The geographic distribution map of *S. marianum*.

*Silybum marianum* (L.) Gaertn is an annual or biennial herb of the genus *Silybum* in the family Asteraceae. It is native to the southern Europe, Asia Minor and northern Africa (Morazzoni and Bombardelli, 1995; Marmouzi et al., 2021). We obtained the geographical distribution of *S. marianum* in the world from the GBIF online database (www.gbif.org). (Figure 1). This plant is able to adapt to harsh environments such as cold (zone 8b), drought, salinity (Martinelli, 2019; Papadimou and Golia, 2024).

The achenes of *S. marianum* are bitter taste, cool nature, and have the effects of clearing heat, protecting liver and gallbladder. It can be utilized for the treatment of jaundice, damp heat of the liver and gallbladder, as well as other related conditions (Committee, 2020; Yu et al., 2023). The chemical constituents found in these achenes mainly consist of flavonolignans, terpenoids, and oil, with flavonolignans representing the largest proportion (Pferschy-Wenzig et al., 2023). Silymarin is a group of flavonolignans including silybin, isosilybin, silydianin, and silychristin, which are formed through the dehydration-condensation process of dihydroflavonols and phenylpropanoid derivatives to produce flavonolignans compounds (Biedermann et al., 2014). Silymarin exhibit hepatoprotective, anticancer, antioxidant, anti-inflammatory, hypoglycemic, neuroprotective, cardioprotective, and immunomodulatory effects (Karimzadeh et al., 2024; Ma et al., 2024). *S. marianum* has been used as a medicinal plant for thousands of years and was first recorded for use in liver protection and venomous snake bites (Morazzoni and Bombardelli, 1995). With the development of modern medicine, the mechanism of action of silymarin has become clearer. Silymarin can resist liver cell fibrosis, protect the liver cell membrane, and promote the repair or regeneration of liver cells when used as a hepatoprotective drug (de Avelar et al., 2023).

When utilized as an anti-cancer medicines, silymarin can block the cell cycle of tumor cells, typically in the G1 phase, and induce apoptosis through multiple mechanisms (Faixová et al., 2023; Mao et al., 2023). As research and applications of active ingredients like silymarin continue to expand and gain traction, traditional extraction methods are struggling to keep up with demand (Yang et al., 2020). The use of medicinal plants for multiple purposes can result in higher economic returns. In addition to medicinal use, *S. marianum* can also be used for oil extraction, a raw material for protein powder, animal fodder, and honey, which making *S.marianum* highly versatile and enhancing economic value (Bencze-Nagy et al., 2023).

With the continuous deepening of research on this medicinal plant, more and more effective components have been found and isolated. In addition to medicinal use, more utilization value of plants is also constantly being discovered. However, there are still some omissions in the research of *S*. *marianum*. For example, many of the active ingredients needed to be proven in *S*. *marianum* that have not yet been isolated and it is necessary to explore the biological functions of various active ingredients, meanwhile, it is absolutely imperative that using science and technology to develop higher value-added. In this review, the botany, phytochemistry, pharmacology, synthetic biology and comprehensive utilization of *S*. *marianum* in recent years are summarized comprehensively and deeply, which provides novel idea for further clinical application and resource development of *S*. *marianum*.

## 2 Methodology

For this review, a comprehensive literature search was conducted up to 24 February 2024. Most of the literature research was conducted through the following five online scientific databases: PubMed, Google Scholar, SciFinder, Web of Science and CNKI. The keywords used to search were: “Silybum marianum,” “Phytochemistry,” “Pharmacology,” “Synthetic biology,” and “Comprehensive utilization”. In addition, the names of all phytochemical compounds were used in the search. The review also included results from the *Flora of China* (http://www.iplant.cn), and relevant conference proceedings written in English and Chinese. And the chemical structures were accurately depicted using the KingDraw software.

## 3 Botanical characteristics

**FIGURE 2:**
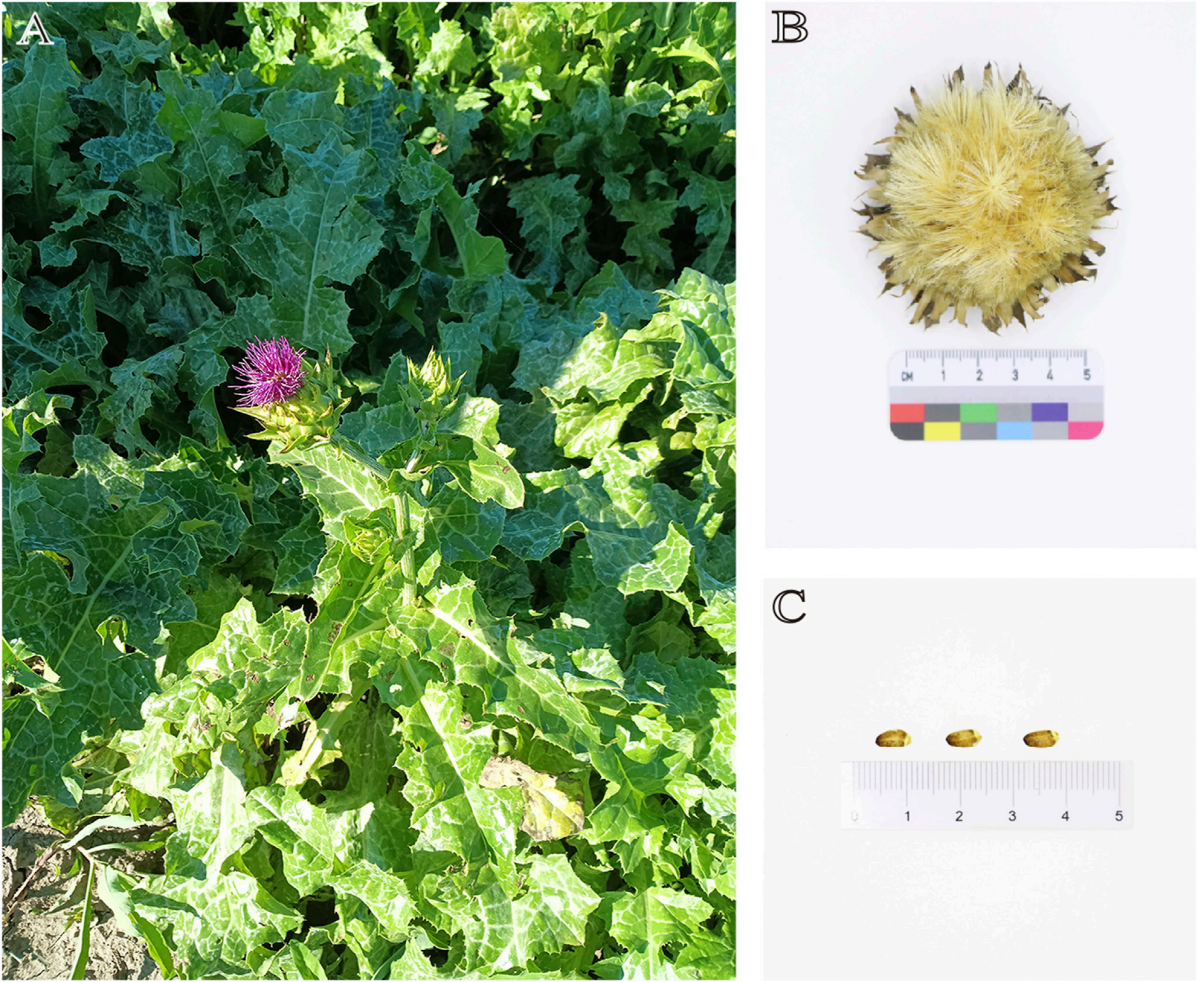
**(A)** The plant of *S. marianum*; **(B)** Dried flower of *S. marianum*; **(C)** Achenes of *S. marianum*.

*S. marianum* is an annual or biennial herbaceous plant with a height of 1–2 m (Figure 2). The stems are erect, striped, multi-branched, with sparse fine hairs or hairless. The rosette basal leaves and the lower stems and leaves have petioles. The leaf shape is oval or inverted lanceolate, about 0.5 m long and 0.3 m wide, and the leaf margin shape is plumose shallow lobes, deep lobes or full lobes; the stems and leaves in the middle and upper parts are small, the leaf shape is lanceolate or long ovate, the leaf margin is pinnate shallow crack and the edge is shallow wavy round tooth crack, the base is gradually pointed and heart-shaped, the upper stems and leaves are small, undivided, lanceolate, and the base amplexicaul shows heart-shape. Variegated green and white are glabrous, the texture of the leaves is thin, and the edges have hard yellow needles with about 5 mm long. he plant has a large capitulum, and the bracts are 3–5 cm long, spherical or ovoid. The middle and outer layers of the entire bracts are oval to lanceolate, with needles on the top and the edge, and no needles on the edge of the base. The upper part may have hard attachments, and their edges and bases have sharp spines, about 1–2 mm long, and the sharp spines at the top are about 5 mm long; the bracts of the inner layer are lanceolate, about 25–30 mm long. Bracts have no acicular edges, no apical attachments, and apex is pointed. The whole bract does not grow fluff, and the texture of the middle and outer bracts are hard and leathery. The flowers are mainly red-purple, with a small amount of white, about 30 mm long, and the thin tube section is about 20 mm long. The filament is thick and short. The achenes are flattened, brownish, and finely ellipsoidal, about 6–8 mm long and 2–3 mm wide, with dark brown spots or stripes. The upper part of the achenes have a edge, which has no serrations. The flowering and fruiting period is from May to August.

## 4 Phytochemistry

### 4.1 Flavonoids

**FIGURE 3:**
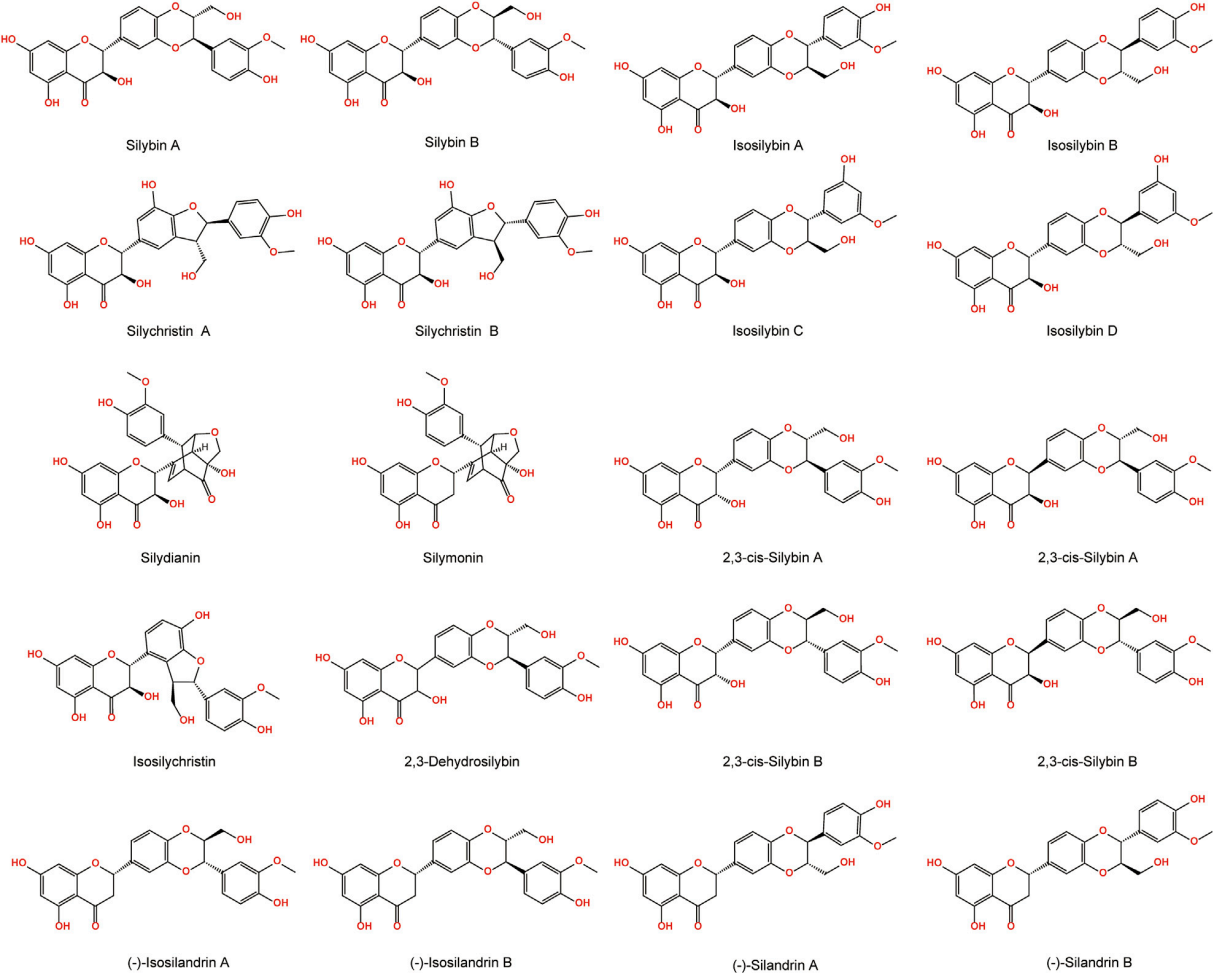
Chemical structures of the flavonolignans constituents of *S. marianum*.

Silymarin is a mixture of several flavonolignans including silybin, dehydrosilybin, isosilybin, silydianin, and silicristin, among which silybin having the highest content of these compounds (Csupor et al., 2016) (Figure 3). In 1968, Wagner et al. were the first to isolate a pharmacologically active silymarin constituent from achenes of *S. marianum* (Wagner et al., 1968). In the same year, Pelter et al. first isolated silybin (Pelter and Hänsel, 1968). Silicristin, the second component of *S. marianum* was discovered by Wagner et al., in 1971 (Wagner et al., 1971) and its structure was determined 3 years later (Pelter et al., 1977). In 1976, silydianin was reported by Wagner et al. (Wagner et al., 1976). The regional isomer of silydianin, isosilydianin, was first reported by Arnone et al., in 1979 (Arnone et al., 1979). Subsequently, isosilicristin was reported by Kaloga et al., in 1981 (Kaloga, 1981). In 2003, the corresponding isomers of silybins A, silybins B, isosilybin A, and isosilybin B were isolated. Achenes of *S. marianum* contain silicristin A, silicristin B, 2,3-cis-silybin A, 2,3-cis-silybin B, and more than 20 types of flavonolignans. In addition, *S. marianum* contains flavonoids component such as quercetin, saccharin, naringenin, flavanolignans, kaempferol, and dihydropyran-4-one, among others (Begum et al., 2010).

### 4.2 Oil compounds

**FIGURE 4:**
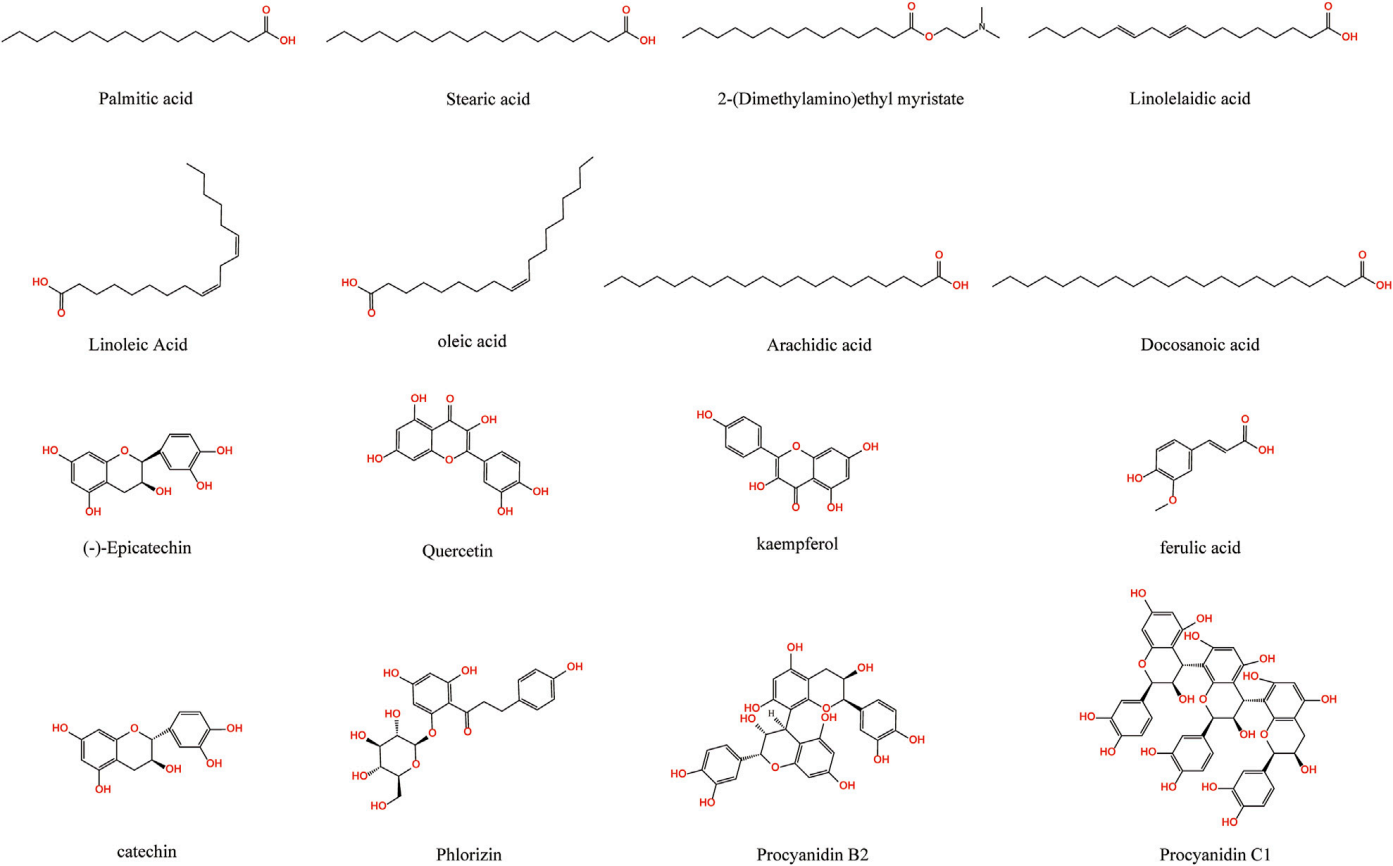
Fatty acids and phenolic acids in *S*. *marianum*.

Oil compounds in achenes of *S. marianum* account for a large proportion, about 25%∼30%, of which linoleic acid content is about 46.46% ± 0.26% (Chen and Wang, 1998; Zhang, 2011) (Figure 4). There are some differences in the types and contents of fatty acids in *S. marianum* of different producing areas. For instance, the content of grease in achenes of *S*. *marianum* cultivated in Egypt is about 35%. In addition, behenic acid and arachidic acid were isolated. Stearic acid and myristic acid have also been isolated from the achenes of *S*. *marianum* that produced in India (Zarrouk et al., 2019).

### 4.3 Others

Achenes of *S. marianum* also contains about 20% protein and 30% starch. *S. marianum* also contains a small amount of triterpenoids, dozens of polyacetylene and polyolefin compounds, alkaloids and sterols (Chen and Wang, 1998; Begum et al., 2010; MacDonald-Ramos et al., 2021; Javeed et al., 2022).

## 5 Pharmacological activity

### 5.1 Liver effects

#### 5.1.1 Treatment of liver damage caused by Alcohol

Alcoholic liver disease often presents oxidative stress, inflammation, liver injury, and liver fibrosis (Stolf et al., 2017). Alcohol causes an imbalance in hepatic lipid synthesis, loss, and degradation, leading to the formation of fatty liver (Ball and Kowdley, 2005). Alcohol can produce the more toxic acetaldehyde through its metabolism. These two substances degrade the ferritin proteins in rat hepatocytes, thereby releasing free Fe^2+^. This process inhibits the expression of the Glutathione peroxidase 4 protein, reduces mitophagy, and increases iron death. Silymarin reduces the level of malondialdehyde, reactive oxygen species (ROS), Fe^2+^, and maintains a normal number of mitochondria. This helps to reduce hepatocyte apoptosis and protect the liver (Rambaldi et al., 2007).

#### 5.1.2 Protecting liver cell membranes

Silymarin protects liver cell membranes by inhibiting lipid peroxidation, which maintains fluidity. It also prevents the specific binding of mycotoxins, such as ghost penitoxin peptide and α-goitrogens, to receptors on liver cell membranes. This inhibits damage to cell membranes, restrains transmembrane transport of toxins, and blocks hepatic-intestinal recycling of toxins, thus enhancing the resistance of the liver cell membranes (Saller et al., 2008; Abenavoli et al., 2010). Studies have shown that silymarin can restore the increase in superficial fluidity of hepatic microsomal and mitochondrial membranes induced by carbon tetrachloride, as well as the decrease in deep fluidity (Abenavoli et al., 2018).

#### 5.1.3 Anti-hepatic fibrosis

Liver fibrosis is typically initiated by inflammation of liver tissue and necrosis of liver cells (Saller et al., 2001; Abenavoli et al., 2010). Previous research has demonstrated that pre-collagen type III peptide (PIIIP) is a reliable indicator of the severity of hepatic fibrosis. Silymarin treatment decreases serum PIIIP levels. Silymarin may inhibit hepatic fibrosis by reducing reactive oxygen species activity and mitigating hepatocellular injury and liver tissue inflammation (Abenavoli et al., 2018; Zhai et al., 2019).

#### 5.1.4 Promoting the repair and regeneration of hepatocytes

**FIGURE 5:**
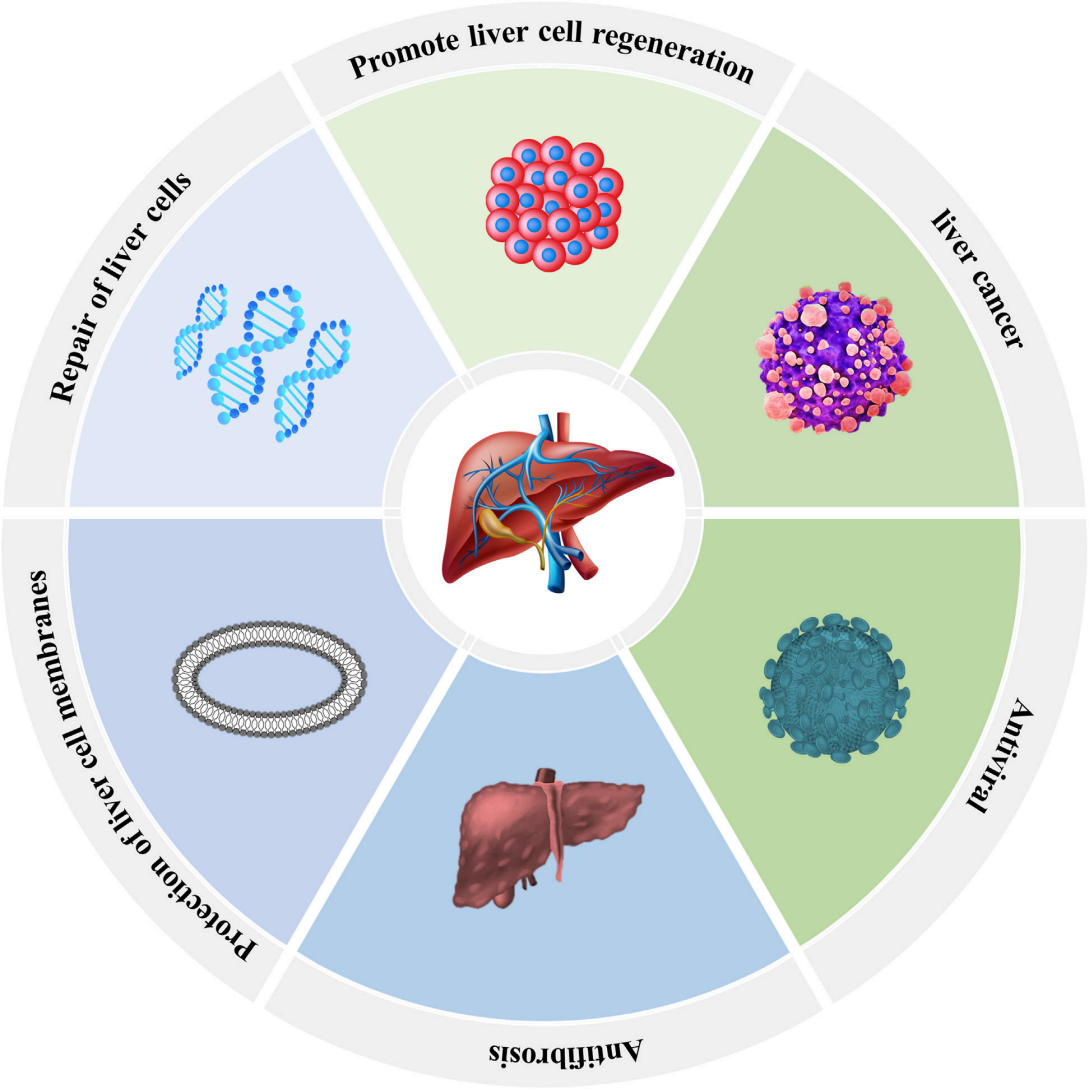
Pharmacological effects of *S. marianum* on the liver.

Silymarin specifically binds and activates the estradiol receptor in hepatocytes, which enhances the activity of RNA polymerase I in the nucleus and promotes the transcription of ribosomal RNA. An increase in the number of ribosomes promotes the synthesis of structural proteins and enzymes and indirectly promotes DNA synthesis, contributing to the repair and regeneration of hepatocytes (Flora et al., 1998) (Figure 5).

### 5.2 Anti-cancer effects

#### 5.2.1 Hepatocellular carcinoma

Hepatocellular carcinoma is the most common primary cancer and is one of the leading causes of cancer-related deaths worldwide. It can occur due to various reasons, including alcohol consumption, fatty liver disease, chronic liver disease, and viral hepatitis B or A. Hepatocellular carcinoma develops gradually through genomic alterations that alter the hepatocyte morphology. The cells progress from an intermediate form to cancerous cells over time (Marengo et al., 2016).

Research has shown that silymarin can effectively decrease the rat liver Lipoperoxides (LOP) content, increase the liver Glutathione (GSH) content, and protect the liver from oxidative stress (Ramasamy and Agarwal, 2008). Additionally, the reduction in LOP content stabilizes the permeability of the liver membrane, which helps to maintain the antioxidant capacity of the liver and the level of ribosomal RNA synthesis (Kwon et al., 2013). Silymarin significantly reduces the expression of the proliferation marker Proliferation cell nuclear antigen (Ki-67) in liver tissue. It also prevents the elevation of serum tumor markers such as alpha fetoprotein and carcinoembryonic antigen in rats and inhibits the occurrence of hepatocellular carcinoma (Pradhan and Girish, 2006). Overexpression of Hepatocyte growth factor (HGF) and Cellular-mesenchymal epithelial transition factor (c-Met) occurred after stimulation of hepatocellular carcinoma cells (HCC) with Tetrachloromethane and Diethylnitrosamine. The combination of HGF and c-Met leads to the phosphorylation of downstream effectors, such as PI3K/Akt, RAS/MAPK, nonreceptor tyrosine kinase, and Focal adhesion kinase, which promotes the survival, proliferation, invasion, and metastasis of HCC (Han et al., 2019). Silymarin inhibits the extracellular binding of HGF to c-Met by downregulating c-Met on the cell membrane and inhibiting HGF expression. This leads to inhibition of tumor cell growth, proliferation, metastasis, and other processes (Lau et al., 2016).

Overexpression of PI3K/Akt induces intrahepatic metastasis in hepatocellular HCC and vascular invasion (Rosário and Birchmeier, 2003). PI3K is converted from phosphatidylinositol bisphosphate to phosphatidylinositol trisphosphate through its binding of Phosphatidylinositol-3-kinase (PI3K) to articulin via SH2/SH3 structural domains. Akt is then phosphorylated, which in turn phosphorylates several cellular target proteins, including mTOR and glycogen synthase kinase 3, ultimately promoting cell cycle progression (Shaw and Cantley, 2006).

**FIGURE 6:**
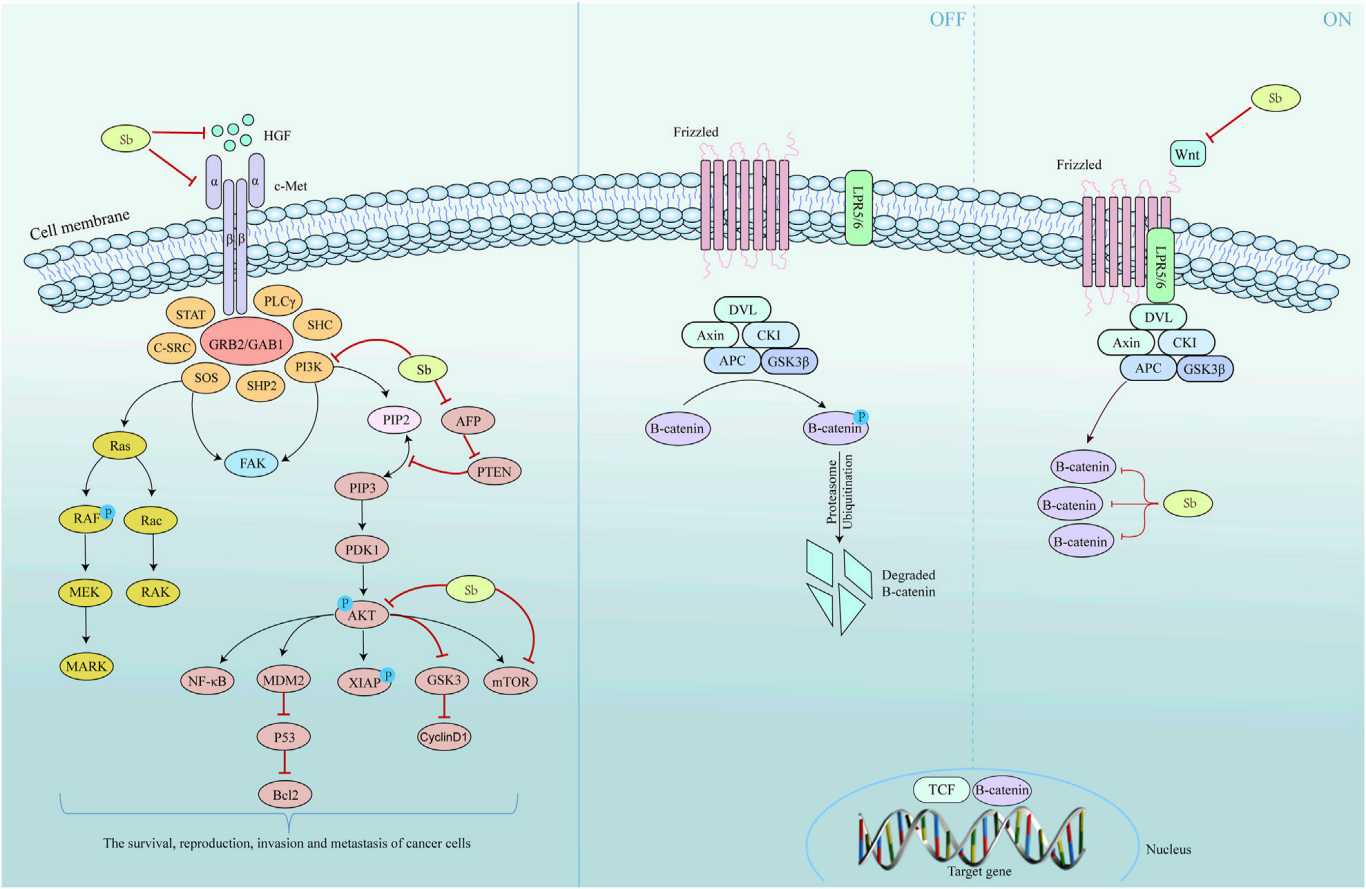
Mechanism of *S. marianum* in the treatment of liver cancer. Left is the HGF/c-Met and the PI3K/Akt/mTOR pathway, and the Wnt/β-catenin pathway on the right.

Activation of the PI3K/Akt/mTOR pathway contributes to HCC progression in liver fibrosis and hepatocellular carcinoma cells. Silymarin binds to enzymes of the PI3K family, thereby inhibiting the activation of the Akt and mTOR families (Peng et al., 2017). The production and development of HCC are also related to the dysregulation of Wnt/β-catenin signaling pathway (Zhang M. et al., 2018). Activation of the Wnt/β-catenin signaling pathway inhibits the degradation of β-catenin, leading to its cytoplasmic accumulation and translocation to the nucleus. β-catenin also complexes with transcription factors such as TCF/Lef, which can activate downstream target genes associated with malignant tumor development, such as Recombinant Protein, Cancer-myc, and cyclin D, thereby stimulating cancer cell proliferation and metastasis (Dahmani et al., 2011). Silymarin inhibits the proliferation of HCC by reducing Wnt mRNA expression and downregulating the level of β-catenin protein (Nusse and Clevers, 2017). (Figure 6)

#### 5.2.2 Gastric cancer

This research found that silymarin exhibited different mechanisms of action in three types of gastric cancer cells, namely, SGC-7901, BGC-823, and HGC-27 (Zhang et al., 2013b). In SGC-7901 cells, silymarin increased the expression of p53 and p21 and decreased the expression of Cyclin-dependent kinases 1 (CDK1). This resulted in the reduction of the CDK1-Cyclin B1 complex, causing the cells to stagnate in the G2/M phase. Additionally, silymarin induces apoptosis in SGC-7901 cells independent of the caspase pathway. In BGC-823 cells, silymarin had a limited effect, slightly reducing CDK1 and activating cysteinyl aspartate specific proteinase 3 (caspase 3) to a small extent. Therefore, silymarin had a weak effect on the induction of apoptosis in BGC-823 cells. Silymarin significantly inhibits the proliferation of HGC-27 cells, reduces the expression levels of CDK1 and Cyclin B1, causes G2/M phase cycle arrest, and activates caspase 3 to cause Poly ADP-ribose polymerase cleavage as well as caspase 8 and caspase 9, ultimately leading to apoptosis of tumor cells (Cartee et al., 2001).

#### 5.2.3 Lung cancer

Silymarin targets several cytokines, including Interferon gamma (IFN-γ), Interferon beta-1 (IF-1β), and Tumor necrosis factor (TNF-α), by binding to Signal transducer and activator of transcription 3 (STAT3). This inhibits the expression of vascular endothelial growth factor by regulating COX-2 and Inducible nitric oxide synthase (Singh et al., 2006; Chittezhath et al., 2008). Silymarin can reduce the size and number of lung cancer cells through its anti-angiogenic activity. This is achieved by decreasing the production of cytokines in tumor-associated macrophages and inhibiting the activation of NFκB and STAT3 in lung cancer cells (Tyagi et al., 2009; Wang et al., 2020a; Verdura et al., 2021).

#### 5.2.4 Kidney cancer

Silymarin has been shown to inhibit the growth of SN12K1 kidney cancer cells (Hii et al., 1998). At low concentrations, silymarin affected renal cancer cell morphology and inhibited DNA synthesis. At high concentrations, silymarin increased the release of lactate dehydrogenase (LDH) and induced apoptosis and necrosis of SN12K1 renal cancer cells. The effects of silymarin on renal cancer cells were studied *in vivo* using a model created by transplanting the renal cancer cells into healthy animals. After comparing the results with those of the control group, silymarin administration was found to reduce both the weight and size of the tumors (Cheung et al., 2007).

#### 5.2.5 Bladder cancer

Silymarin effectively induced the expression of Cip1/p21 and Kip1/p27 proteins while decreasing the expression of CDK2, CDK4, and CDK6 and the cell cycle proteins Cyclin D1, Cyclin D3, and Cyclin E. It also increases the mutual binding of Cyclin-dependent kinases inhibitors (CDKI) and CDK, inhibits the kinase activity of CDKs, and ultimately arrests the cell cycle of bladder cancer in the G1 phase (Singh et al., 2002). Furthermore, increased doses of silymarin can decrease the levels of pCdc25c, Cdc25c, pCdc2, Cdc2, and Cyclin B1 proteins in TCC-SUP tumor cells, resulting in cell cycle arrest in the G2/M phase (Tan et al., 2002). Silymarin has varying effects on various bladder cancer cell types TCC-SUP cells can induce G1 and G2/M phase blockage, whereas T-24 cells can only block G1 phase blockage. Silymarin can significantly induce apoptosis in TCC-SUP cells, but the effect is not significant in T-24 cells (Tyagi et al., 2004).

#### 5.2.6 Cervical cancer

The research found that silymarin treatment resulted in a four-fold increase in the number of HeLa cells in the G2/M phase compared to that in the control group. This suggests that silymarin may inhibit the progression of HeLa cells in cervical cancer and induce apoptosis, as evidenced by the presence of apoptotic precursors, such as cell crumpling. Silymarin downregulated CDK1 and CDK2 protein levels in a concentration-dependent manner and induced apoptosis in HeLa breast cancer cells in a time and concentration-dependent manner (Fan et al., 2011). It can activate the mitochondrial apoptotic pathway, leading to a decrease in B lymphocyte chemoattractant protein levels, release of cytochrome C from the mitochondria into the cytoplasmic matrix, and activation of caspase 9. It can activate the membrane receptor pathway of apoptosis, which upregulates the protein levels of *Fatty Acid Synthase* (*Fas)* gene and *Fas* ligand and activates Caspase 8 (Zhang et al., 2012).

#### 5.2.7 Prostate cancer

Prostate cancer cells diffuse and infiltrate the prostate mesenchyme instead of forming localized tumors due to the secretion of prostate-specific antigens (PSA) into the prostate mesenchyme. This promotes the cleavage of Insulin-like growth factor-binding protein 3 and Insulin-like growth factor 1 (IGF-1), as well as the activation of transforming growth factor b and other growth factors in the extracellular matrix (ECM). These factors promote tumor cell growth and lead to tumor progression (Wang et al., 1997). Silymarin reduced intracellular and secreted PSA levels in human prostate cancer LNCaP cells and inhibited dihydrotestosterone-induced PSA production and cell growth. It can also inhibit malignant tumors by overexpressing cell-cycle proteins. Silymarin significantly decreased the levels of the cell cycle proteins D1, CDK4, and CDK6, leading to reduced kinase activity. Additionally, there was a significant increase in Cip1/p21 and Kip1/p27 (Poluha et al., 1996), which led to an increase in their binding to CDK2. In turn, this resulted in a significant reduction in CDK2 and cyclin E kinase activities, ultimately causing tumor cells to arrest at the G1 phase and inhibiting the growth of LNCaP cells (Mueller et al., 1997). Aldehyde dehydrogenase 1 family, member A1 (ALDH1A1) is an aldehyde oxidase that can be targeted by silymarin for the treatment of prostate cancer. It regulates the synthesis of trans- and 9-cis-retinoic acid, which inhibits the proliferation and differentiation of tumor-promoting macrophages stimulated by cancer cells. In addition, it also acts as an oncogene in prostate cancer (Yoshida et al., 1992). Studies have shown a positive correlation between ALDH1A1 expression in prostate cancer tissue and Recombinant retinoic acid receptor alpha (RARα) and Erythroblastosis-twenty six 1 (Ets1). RARα can bind to the Ets1 promoter and induce the expression of Ets1 mRNA and protein in cancer cells (Raouf et al., 2000). Ets1 affects the degradation of the extracellular matrix, which can facilitate the metastasis of tumor cells. The overexpression of Ets1 is closely related to the deterioration of prostate cancer (Li et al., 2012). ALDH1A1 promotes the invasion and metastasis of prostate cancer by activating RARα, which further activates Ets1. Silymarin inhibits the expression of ALDH1A1 in prostate cancer, which in turn inhibits the further activation of RARα and Ets1. Thus inhibiting the growth and metastasis of prostate cancer (Nazir et al., 2019).

#### 5.2.8 Skin cancer

There was a strong correlation between elevated P53 levels and apoptosis induction in chronic ultraviolet radiation b (UVB)-exposed skin and tumors treated with silymarin (Matsumura and Ananthaswamy, 2004). P53 induces apoptosis in human keratinocytes at high doses of UV irradiation and activates the UV-induced repair of DNA damage at low doses of irradiation (Cotton and Spandau, 1997). p53 promotes cell repair and survival while promoting apoptosis. Similarly, silymarin protects cells from UV-induced apoptosis during acute injury and promotes apoptosis during chronic UV-induced injury (Dhanalakshmi et al., 2004). Silymarin upregulates Kip1/p27 and Cip1/p21 expression in tumors, which decreases the protein levels of CDK2, CDK4, Cyclin E, Cyclin A, and Cyclin D1, ultimately leading to tumor cell cycle arrest and reduce proliferation (Fotedar et al., 2004). Treatment with silymarin results in a significant increase in the phosphorylation of extracellular regulated protein kinases, JNK1/2, and p38 in the tumor samples. Activation of ERK induces cell cycle arrest by inducing the expression of the CDK inhibitors Cip1/p21 and Kip1/27, which activate the apoptotic response of JNK1/2 and p38 kinases. In HaCaT cells, p38 is activated by the release of cytochrome c into the cytosol, which in turn activates caspase-3 to mediate apoptosis in UVB (Polyak et al., 1994). *Survivin* molecular antagonists have been shown to induce caspase-dependent cell death, enhance apoptosis, and exhibit anticancer activity *in vivo*. Additionally, silymarin has been shown to reduce *Survivin* levels in tumors. In conclusion, silymarin can inhibit skin cell carcinogenesis by inhibiting DNA synthesis, cell proliferation, blocking the cell cycle, and inducing apoptosis (Bachelder et al., 1999).

#### 5.2.9 Breast cancer

Silymarin inhibites both MCF-7 and SK-BR-3 breast cancer cell lines A low dose of silymarin can strongly inhibit MCF-7, leading to cell autophagy and apoptosis by down-regulating the expression of Estrogen Receptor α (ERα) in MCF-7 cells (Zheng et al., 2017). Additionally, silymarin upregulates the expression of ERβ, which induces apoptosis via the mitochondrial pathway. However, silymarin has shown to only weakly inhibit the growth of SK-BR-3 cells. Studies have suggested that this may be related to the protein tyrosine kinase molecule Human epidermal growthFactor receptor 2 (Her-2) in breast cancer cells (Templeton et al., 2014). The expression level of Her-2 is considered an important indicator of the degree of malignancy and prognosis of breast cancer (Kurokawa et al., 2000). The expression of the Her-2 molecule has been found to increase the proliferation of SK-BR-3 breast cancer cells and decrease their sensitivity to silymarin (Hermanto et al., 2001).

#### 5.2.10 Colon cancer

Silymarin inhibits the growth of colon cancer cells by blocking the cell cycle via multiple mechanisms. Specifically, it upregulates kip1/p27, a key member of CDKI that counteracts proliferative signals. Tumor cells with low or no CDKI expression exhibited uncontrolled growth. Silymarin increased the mRNA and protein expression of kip1/p27. Additionally, it can increases the protein expression of Cip1/p21 without relying on the regulation of the p35 oncoprotein. It also blocked the G1 phase of HT-29 colon cancer cells (Sherr, 1996). Cdc25C acts as a mitotic activator by dephosphorylating cdc2/p34. The activity of cdc2/p34 kinase is enhanced in human cancers. Higher doses of silybin reduce cdc2/p34 kinase activity, decrease the protein expression of cdc25C, cdc2/p34, and cyclin B1, and block HT-29 colon cancer cells in the G2 phase (Graves et al., 2000). Longer treatment with silymarin also causes HT-29 tumor cells to undergo apoptosis independent of the caspase pathway (Chinni et al., 2001). (Table 1).

**TABLE 1:** Anti-cancer cellular pathways involved in *S. marianum*.

Cancers	Cells	Pathways	Ref
Hepatocellular carcinoma	HCC	HGF/c-Met、Wnt/β-catenin	Yassin et al. (2022)
Gastric cancer	SGC-7901	P53/p21、Caspase、PI3K/Akt/mTOR	Mi et al. (2022)
Gastric cancer	BGC-823	CDK1、Caspase	Fallah et al. (2021)
Gastric cancer	HGC-27	CDK1、Cyclin B1、Caspase	Mi et al. (2022)
Lung cancer	NSCLC	NFκB、JAK/STAT	Verdura et al. (2021)
Kidney cancer	SN12K1	P53/p21、TGFβ、PI3K/Akt/mTOR	Yassin et al. (2021)
Bladder cancer	TCC-SUP	Cip1/p21、Kip1/p27、CDK	Gándara et al. (2014)
Bladder cancer	T-24	Cip1/p21、Kip1/p27、CDK	Gándara et al. (2014)
Cervical cancer	HeLa	PI3K/Akt/mTOR、Caspase	You et al. (2020)
Prostate cancer	LNCaP	Cip1/p21、Kip1/p27	Wu et al. (2023b)
Breast cancer	MCF-7	ER	Zheng et al. (2016)
Breast cancer	SK-BR-3	HER-2	Zheng et al. (2016)
Colon cancer	HT-29	Cip1/p21、Kip1/p27	Fallah et al. (2021)
Skin cancer	HaCaT	Kip1/p27、Cip1/p21、ERK、JNK	Yassin et al. (2022)

### 5.3 Antioxidant effects

Silymarin enhances the antioxidant capacity of the body via several mechanisms. First, it directly scavenges the free radicals. Secondly, it inhibits ROS-generating enzymes, thereby preventing free radical production. Additionally, silymarin activates a series of antioxidant enzymes, such as NF-E2-related factor 2 and Nuclear factor kappa B (NF-κB), to maintain an optimal redox balance in cells. Silymarin can activate the molecules responsible for protecting organisms, such as Heat shock protein, Thioredoxin, and sirtuins, providing additional protection during oxidative stress (Surai, 2015). Studies have shown that increased production of reactive oxygen metabolites is a significant cause of sepsis. Free radicals, in addition to causing direct tissue damage, may lead to the accumulation of leukocytes in tissue, which activate neutrophils and cause further damage. This disease causes a systemic inflammatory response that may ultimately progress to systemic multi-organ failure (Sener et al., 2005a; Sener et al., 2005b). Silymarin reduces oxidative organ damage induced by sepsis by inhibiting neutrophil infiltration, which in turn blocks the release of cytokines such as leukotrienes (Lts) and Interleukin-1 (IL-1) (Toklu et al., 2008). Severe burns can trigger an inflammatory response that damages the affected tissue. This damage can lead to sepsis and multi-organ failure in severe cases (Sayeed, 1998; Schwacha and Chaudry, 2002). Burns results in a significant increase in pro-inflammatory factors TNF-a and LDH, leading to an increase in MDA levels and a decrease in GSH levels in the skin. Silymarin can reverse these effects by inhibiting burn-induced oxidative damage to the skin. Additionally, silymarin can reverse the morphological damage caused by burns on the skin (Toklu et al., 2007). Oxidative stress is considered a crucial mechanism in Doxorubicin (DOX)-induced cardiomyocyte damage. High levels of ROS promote autophagy, whereas low levels inhibit it. DOX causes a slight increase in ROS, and moderate autophagy helps maintain intracellular homeostasis by degrading redundant, aged, and misfolded proteins, releasing energy, or removing damaged organelles (Taghiabadi et al., 2012). This suggests that low ROS production is crucial for the inhibition of DOX-induced autophagy. Additionally, silymarin counteracts myocardial injury by activating IL6ST/JAK2/STAT3, which helps eliminate ROS and restore autophagy (Li W. et al., 2022).

### 5.4 Inhibition of NO production

Cytotoxic NO production increases in pathological states of liver damage. Excessive NO levels can cause hypoxemia and hyperdynamic cycles. Additionally, NO reacts with O_2_ to produce nitrite, an unstable and weak acid that decomposes into a strongly toxic NO_2_ group. Silymarin inhibits the production of NO by Kupffer cells, thereby reducing the amount of NO_2_ and other toxic groups that protect the body (Chittezhath et al., 2008).

### 5.5 Anti-inflammatory

The anti-gastric ulcer activity of silymarin is attributed to its inhibition of enzymatic peroxidation in the lipoxygenase pathway, which inhibits leukotriene synthesis (Alarcon de la Lastra et al., 1992). Silymarin has free radical scavenging activity. It can also regulate arachidonic acid cascade and inhibit the production of Prostaglandins and Leukotrienes, thus effectively inhibiting the development of arthritis (Gupta et al., 2000).

### 5.6 Immunomodulatory effects

Research on alcohol-induced liver disease revealed that ethanol metabolism generates acetaldehyde adducts that activate the immune system as foreign antigens. This can lead to an increase in the number and activity of Cytotoxic T lymphocyte (CTL) and Natural killer cells (NK) in the body, thereby exacerbating immune damage to hepatocytes (Yasuda et al., 1999). Following silymarin treatment, there was a decrease in the number of CTL and NK cells in the blood, as well as a reduction in their activity. This suggests that the drug possesses immunomodulatory properties. Additionally, studies have shown that silymarin can inhibit the proliferation of CD4 cell lymphocytes and the production of IL-2 and IFN-γ in mice (Gharagozloo et al., 2010).

### 5.7 Neuroprotective effects

Silymarin may serve as a neuroprotective agent for treating various neurological disorders such as Alzheimer’s disease, Parkinson’s disease, and cerebral ischemia (Borah et al., 2013). Alzheimer’s is characterized by cognitive impairment and the deposition of extracellular amyloid fibrils in senile plaques. Silymarin attenuates these symptoms in antibody-induced animal models of Alzheimer’s disease. Silymarin administration significantly improved cognitive abnormalities, particularly memory impairment, and significantly reduced extracellular amyloid fibrillar deposition in senile plaques (Lu et al., 2009). Parkinson’s disease is characterized by the loss of dopaminergic neurons in the dense part of the substantia nigra and abnormal motor behavior. According to a previous research, silymarin has been shown to significantly increase dopamine and serotonin levels in the hippocampal and cortical regions and inhibit monoamine oxidase-b. This suggests that silymarin counteracts dopamine loss in patients with Parkinson’s (Singhal et al., 2011).

### 5.8 Treatment of insulin resistance

Insulin resistance refers to the weakening of the physiological role of insulin in the body, and obesity is often the main cause of insulin resistance, which in turn will put obese people in a state of chronic inflammation (MacDonald-Ramos et al., 2024). Insulin resistance can also lead to an increase in blood sugar, and the body secretes more insulin to maintain normal blood sugar levels, leading to hyperpancreatic islet emia and the eventual development of diabetes mellitus type 2 (MacDonald-Ramos et al., 2021). Silymarin’s excellent anti-inflammatory and antioxidant stress effects can be used in IR treatment to alleviate the adverse effects of diseases on the body and prevent the occurrence of diabetes. A number of studies have shown that insulin resistance is effectively inhibited after treatment with a certain dose of silymarin administered to an animal model of insulin resistance, which is achieved by restoring the IRS-1/P13K/Akt pathway and blocking the phosphorylation of c-Jun N-terminal kinase (JNK) and inhibitor of kappa B kinase (Zhang et al., 2013a; Li et al., 2015; Guo et al., 2016).

### 5.9 Treatment of diabetes

**FIGURE 7:**
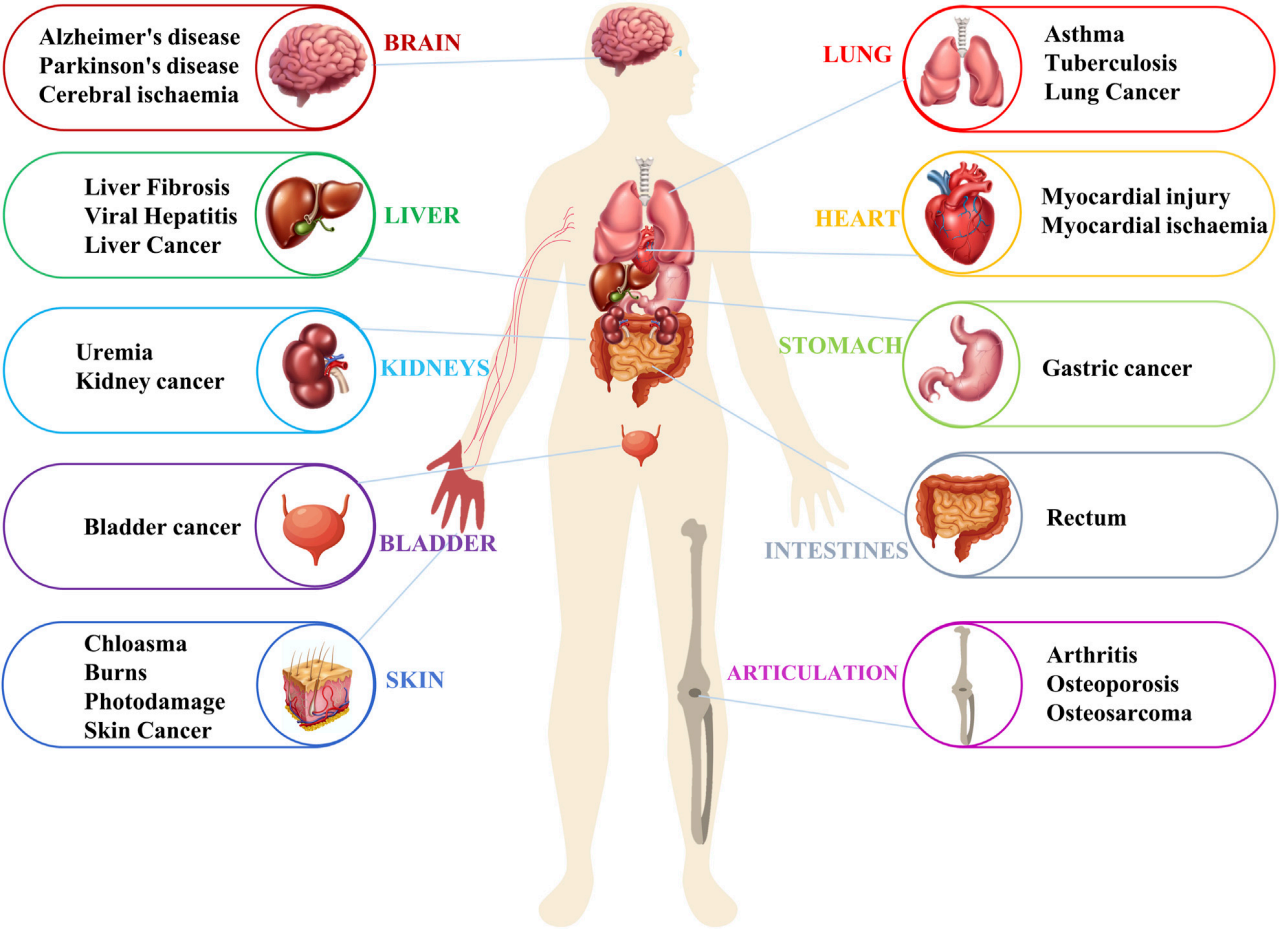
Pharmacological effects and organs of action of *S. marianum*.

Diabetes mellitus is a prevalent metabolic disorder with multiple causes resulting from insufficient insulin secretion or defective insulin action (Stolf et al., 2017). Hyperphagia, polydipsia, polyuria, and weight loss are the common symptoms of diabetes. Failure to control blood glucose levels in a timely manner can result in more than 100 complications, including nephropathy, neuropathy, impaired healing, oxidative stress, cataracts, hepatotoxicity, and cardiomyopathy. In severe cases, it can lead to multi-organ damage and organ failure, such as diabetic end-stage renal failure (Alberti and Zimmet, 1998; Conserva et al., 2016). Silymarin has been shown to be effective in treating alloxan-induced diabetes in rats (Soto et al., 2010). It also increased the activity and expression levels of Superoxide dismutase, GSH peroxidase, and Catalase in the pancreas of diabetic rats. This mechanism of action may be related to the activation of the promoter regions of these enzymes by flavonolignans (Soto et al., 1998; Soto et al., 2003). Additionally, silymarin improved pancreatic morphology and endocrine function in diabetic rats and repaired damaged kidney tissue (Soto et al., 2004). Patients with end-stage diabetic nephropathy exhibit significant thiol deficiency, which is directly related to a decrease in T cell activity and an increase in the synthesis of TNF-α. This promotes ROS production by neutrophils. Silymarin ameliorated or reversed these symptoms (Dietzmann et al., 2002). (Figure 7) (Table 2).

**TABLE 2:** Pharmacological effects and mechanisms of silymarin.

Pharmacological effects	Extracts/Compounds	Types	Animal/cell	Dosage	Effects	Ref
alcoholic liver disease	Silymarin	*In vivo*	C57BL/6 mice	60 mg/kg	Reducing alcohol-induced hepatic steatosis by upregulating the LKB1/AMPK/ACC signaling pathway	Feng et al. (2019)
	Silybin	*In vivo*	SD rat	100 mg/kg	Inhibition of mitochondrial division reduces apoptosis rate	Song (2023)
	Silybin	*In vivo*	C57BL/6 mice	100 mg/kg	Blocking alcohol-induced oxidative stress and lipid peroxidation	Wang (2023)
nonalcoholic fatty liver disease	Silybin	*In vivo*	C57BL/6 mice	50 or 100 mg/kg/day	Attenuating ER stress to regulate P450s activity	Wu et al. (2023a)
	silibinin	*In vivo*	C57BL/6 mice	5 mg/kg	abolished oxidative stress, and inhibited PARP activation thus restoring the NAD⁺ pool	Salomone et al. (2017)
	Silybin	*In vivo*	BALB/c mice	100 mg/kg/day	Combat obesity caused by the whole body	Ma et al. (2024)
	Silibinin	*In vivo*	SD rat	100 mg/kg	Improved liver oxidative stress and inflammation	Zhang et al. (2013a)
	Silybin	*In vivo*	C57BL/6 mice	40 or 80 mg/kg/day	Inhibits inflammation and reduces the expression of CYP3A	Zhang et al. (2021)
	Silybin	*In vivo*	C57BL/6 mice	50 or 100 mg/kg/day	Regulating lipid disorders	Sun et al. (2020)
viral hepatitis	Silymarin	*In vitro*	Huh7 cell	100–300 μmol/L	Stimulates Jak-Stat pathway and induces IFN antiviral response	Polyak et al. (2007)
	Silymarin	*In vitro*	Huh7.5.1 cell	40、80 or 120 μmol/L	Inhibition of MTP activity, apoB secretion and production of infectious virus particles	Wagoner et al. (2010)
	Silymarin	*In vitro*	PBMC cell	20 or 40 μmol/L	Inhibition of T cell proliferation and proinflammatory cytokine secretion	Morishima et al. (2010)
Liver injury induced by carbon tetrachloride	Silymarin	*In vivo*	Wistar rat	50 mg/kg/day	Reduce liver inflammation, improve liver cell synthesis function	Guo and Yang (2008)
	Silymarin	*In vivo*	C57BL/6J mice	0.2 mmol/kg	Significantly reduced the expression of pro-inflammatory factors in the liver	Xu et al. (2022)
	Silymarin	*In vivo*	SD rat	200 mg/kg	Significantly inhibited transaminase activity and liver fibrosis	Khalil et al. (2021)
Hepatic fibrosis	Silymarin	*In vivo*	Albino rat	300 mg/kg	Exert the stability and antioxidant activity of the membrane	Mukhtar et al. (2021)
	Silymarin	*In vivo*	Wistar rat	50 mg/kg/d	Dmn-induced liver fibrosis can be partially blocked and reversed	Zhao et al. (2006)
Liver cirrhosis	Silybin	*In vivo*	SD rat	25、50 or 100 mg/kg	The expression of nuclear Nrf2 was significantly upregulated	Li et al. (2022a)
Liver cancer	Silybin	*In vitro*	HepG2 cell	68 μmol/L	Downregulated miR92a and inhibited AKT activity in a Pten-dependent manner	Zappavigna et al. (2019)
	Silybin	*In vitro*	HepG2 cell	5 mg/kg	Inhibition of Ki-67 expression, HGF/cMet, Wnt/β-catenin and PI3K/Akt/mTOR pathways, and enhancement of antioxidant defense mechanisms	Yassin et al. (2022)
	Silymarin	*In vitro*	Huh-7 cell	0–4.5 μg/mL	The apoptosis rate of hepatocellular carcinoma cells was increased, and the cycle of hepatocellular carcinoma cells was blocked in G1 phase	Rahnama et al. (2023)
Gastric cancer	Silybin	*In vitro*	BGC-823 cell	25 or 50 μmol	G2/M cell cycle arrest and apoptosis were induced	Zhang et al. (2018b)
	Silymarin	*In vitro*	AGS cell	100 mg/kg	Inhibition of p-ERK and activation of p-p38 and p-JNK to reduce tumor growth	Kim et al. (2019)
	Silybin	*In vitro*	AGS cell	32 μg/mL-1024 μg/mL	Inhibition of NO production associated with TNF-α, IL-6 and IL-10 cytokines	Bittencourt et al. (2020)
Kidney cancer	Silybin	*In vivo*	Wistar rat	5 mg/kg	The apoptotic proteins p53 and caspase-3 were downregulated and the anti-apoptotic mediator Bcl-2 was upregulated	Yassin et al. (2021)
	Silybin	*In vitro*	769-P cell	40、60 or 80 μmol	Wnt/β-catenin signaling was inhibited in an autophagy dependent manner	Fan et al. (2020)
	Silybin	*In vitro*	769-P cell	0–200 μmol	Apoptosis was induced by regulating the mTOR-GLI1-BCL2 pathway	Ma et al. (2015)
Bladder cancer	Silybin	*In vitro*	T24 cell	50、100 or 200 μmol	Interfere with the interaction between Apaf-1 and Hsp70 to increase pro caspase-9	Wei et al. (2024)
	Silybin	*In vitro*	T24 cell	50 μmol	Metastasis is induced by inhibition of EMT	Li et al. (2018)
	Silybin	*In vitro*	T24 cell	10 μmol	Downregulated actin cytoskeleton and PI3K/Akt pathway	Imai-Sumida et al. (2017)
Cervical cancer	Silybin	*In vivo*	BALB/c mice	300 mg/kg	Activation of kinetic protein-associated protein 1 (Drp1) induces G2/M cell cycle arrest	You et al. (2020)
	Silybin	*In vitro*	HDF cell	100 μmol or 200 μmol	The expression of type I and type III collagen in HDFs and KFs was significantly reduced	Choi et al. (2023)
Prostate cancer	Silybin	*In vitro*	C4-2 cell	0–200 μmol	The invasion, migration and EMT of CRPC cells were inhibited	Dan et al. (2022)
	Silybin	*In vitro*	PC-3 cell	3–120 μg/mL	The blocked cells remained in G1 and G2/M phases	Gioti et al. (2019)
Skin cancer	Silymarin	*In vivo*	BALB/c mice	100 mg/kg	Reduce chromosome damage and delay the occurrence of tumor	Karem et al. (2021)
	Silymarin	*In vitro*	A2058 cell	15–125 μg/mL	Significantly reduced IL-6 production in cells	Gjörloff Wingren et al. (2023)
Breast cancer	Silybin	*In vitro*	MDA-MB-231 cell	40、80 or 160 μmol	The expression of Rac1 mRNA was significantly inhibited	Lashgarian et al. (2020)
	Silymarin	*In vitro*	MCF-7 cell	25 or 50 mg/kg	Breast cancer cell proliferation was inhibited by regulating MAPK signaling pathway	Kim et al. (2021)
Colon Cancer	Silybin	*In vitro*	DLD-1 cell	12.5 μmol	Significantly inhibited the growth of tumor cells	Sayyed et al. (2022)
	Silybin	*In vitro*	CaCo-2 cell	5–80 μmol	Increased apoptosis and significantly decreased the expression of pro-inflammatory interleukin and TGF-β genes	Faixová et al. (2023)
Inhibition of nitric oxide production	Silymarin	*In vitro*	mesangial cell	50 μg/mL	The expression of iNOS gene was inhibited in cells	Youn et al. (2017)
Anti-inflammatory	Silybin	*In vivo*	C57 mice	100 mg or 200 mg/kg	Activate the Nrf2 pathway to promote antioxidant action	Wei et al. (2022)
	Silybin	*In vivo*	Wistar rat	150 mg/kg	The expression levels of TNF-α, IL-1β and IL-6 were significantly downregulated	Li et al. (2023)
	Silymarin	*In vivo*	mice	3.125–25 μg/mL	IL-6 and CRP were significantly downregulated	Hanafy and El-Kemary (2022)
Neuroprotective effects	Silybin	*In vivo*	SD rat	25、50 or 100 mg/kg	Inhibition of ER-mediated PI3K/Akt and MAPK pathways	Wang et al. (2002)
Treatment of insulin resistance	Silibinin	*In vivo*	SD rat	100 mg/kg/day	Alleviating steatosis and insulin resistance *in vivo* and *in vitro* by modulating the IRS-1/PI3K/Akt pathway	Zhang et al. (2013a)
	silymarin	*In vivo*	C57BL/6 mice	40 mg/100 g	Ameliorated insulin resistance, dyslipidaemia and inflammation, and reconstituted the bile acid pool in liver of diet-induced obesity	Gu et al. (2016)
Treatment of Diabetes	Silibinin	*In vivo*	SPZF rat	100 or 300 mg/kg	The quality and function of L cells were improved through the ER-mediated antioxidant pathway	Wang et al. (2022)
	Silymarin	*In vivo*	Fischer rat	50 or 100 mg/kg	Reduced liver and pancreas protein damage and creatinine levels	Miranda et al. (2020)

## 6 Progress in the biosynthesis of silymarin

Coniferyl alcohol and taxifolin are precursors of silymarin biosynthesis, and these two substances have certain organ dependence in *S. marianum*. Studies have shown that coniferyl alcohol is distributed in the whole plant of silymarin, while taxifolin is mainly distributed in flowers, pericarp and embryos, which is also related to the accumulation of silymarin in pericarp (Lv et al., 2017).

**FIGURE 8:**
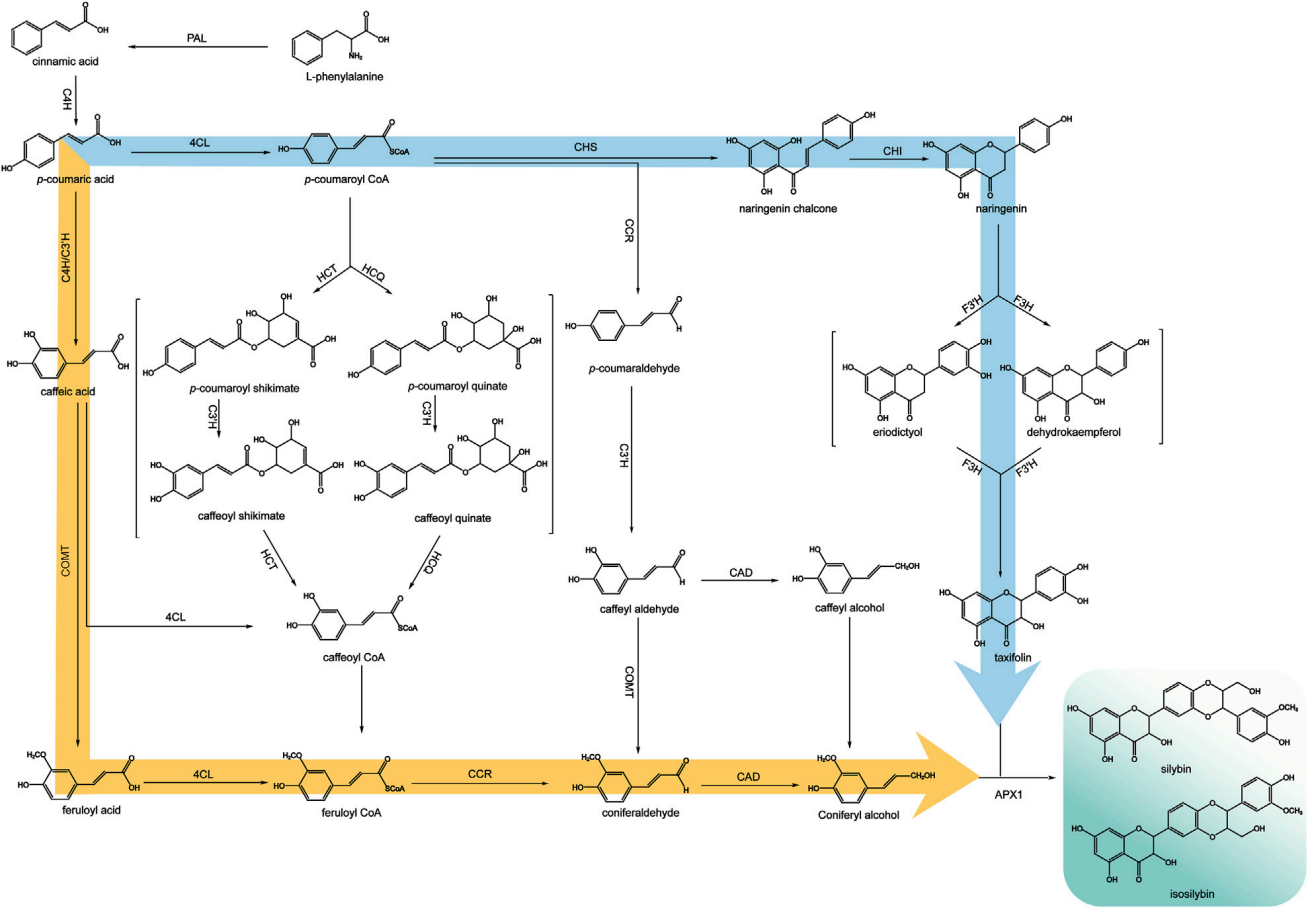
Biosynthetic pathways of silymarin (adapted from (Yang et al., 2020)).

Mechanistic studies have shown that silymarin is produced through the oxidative coupling of coniferyl alcohol and taxifolin, which is formed from the conversion of phenylalanine via the phenylalanine pathway (Martinelli et al., 2017). (Figure 8) This pathway catalyzes the dehydrogenation of phenylalanine by phenylalanine deaminase (PAL) to produce trans-cinnamic acid, which is then catalyzed by cinnamic acid-4-hydroxylase (C4H) to produce p-coumaric acid. Taxifolin is produced by the catalysis of p-coumaric acid to generate p-coumaroyl coenzyme A. Under the catalysis of chalcone synthase (CHS), one molecule of p-coumaroyl coenzyme A is condensed with three molecules of malonyl coenzyme A to generate naringenin chalcone. Naringenin chalcone undergoes successive catalytic reactions of chalcone isomerase (CHI), flavanone 3-hydroxylase (F3H), and flavanone 3′-hydroxylase (F3′H), isomerization, and hydroxylation to finally obtain taxifolin. (Hammerbacher et al., 2019).

Coniferyl alcohol is produced by the action of 4-coumaroyl-coenzyme A ligase (4CL), which catalyzes the conversion of coumaric acid to coumaroyl-coenzyme A. Then this compound is hydroxylated by coumaric acid-3-hydroxylase (C3H) to form caffeoyl CoA. Another way is using coumaroyl-coenzyme A as a substrate, cysteine protease (CST) or quinuclidinic acid hydroxycinnamoyltransferase (CQT) catalyze the production of p-coumaroyl shikimic acid or p-coumaroyl quinic acid. These compounds are then hydroxylated by C3H to produce the intermediates, caffeoyl shikimic acid and caffeoyl quinic acid. Finally, CST or CQT catalyze the conversion of these intermediates to caffeoyl CoA, which is methylated by caffeoyl coenzyme A-O-methyltransferase (CCoAOMT) to produce feruloyl CoA. Because the substrates of C3H, 4CL and methyltransferase are relatively broad, there are other mechanisms from p-coumaric acid to p-coumaroyl CoA, that is, p-coumaric acid is first catalyzed by C3H to generate caffeic acid, and caffeic acid is catalyzed by 4CL to generate caffeoyl CoA and then methylated. Alternatively, caffeic acid can be catalyzed by caffeic acid-O-methyltransferase (COMT) to form the methylated product ferulic acid, which is then catalyzed by 4CL to form feruloyl CoA. Finally, feruloyl CoA is catalyzed by cinnamoyl-CoA reductase (CCR) to produce coniferyl dehyde, which is then catalyzed by cinnamyl alcohol dehydrogenase (CAD) to produce coniferyl alcohol (Jin et al., 2016). Studies have shown that peroxidases, particularly APX1, can couple taxifolin and coniferyl alcohol to generate silybin and isosilybin (Shin et al., 2015; Drouet et al., 2020).

## 7 Comprehensive utilization

### 7.1 Edible oil

Achenes of *S. marianum* have high oil content, usually ranging from 30% to 35%. Silymarin oil is rich in various bioactive compounds including phenolic acids, tocopherols, fatty acids, and phytosterols (Chambers et al., 2017). The oil of achenes in *S. marianum* is also a natural source of vitamin E (Zarrouk et al., 2019). The oil extraction of achenes in *S. marianum* is effective in preventing oxidative stress and restoring normal levels of cholesterol, triglycerides, LDL, and liver markers associated with liver pathology. Therefore, it is often recommended as a beneficial cooking oil (Shin et al., 2015). Cold-pressed oil is produced using a simple method that does not require a high-energy input or added chemicals. This method is more economical and environmentally friendly than traditional refined oils. The oil is purified using water, sedimentation, filtration, and centrifugation. Additionally, the cold-pressed oil technique allows for greater retention of valuable substances in achenes of *S. marianum* (Kalinowska et al., 2022).

### 7.2 Forage

After extracting the active ingredients from achenes of *S. marianum*, a byproduct of residue is produced. Recently, increasing attention has been paid to the use of *S. marianum* residue as animal feed (Stastnik et al., 2020). *S. marianum* residue has starch and protein contents of up to 30% and 20%, respectively, without any toxic side effects. They are rich in amino acids and trace elements (Liu et al., 2012). This is not only conducive to the absorption and supply of energy and protein required for animal growth, but also conducive to the absorption and supply of trace elements. Additionally, it has health benefits, improves immunity, and indirectly reduces the use of antibiotics and other drugs. It has higher added value and can be used as a new high-quality feed for poultry, livestock, and fishery farmin (Chambers et al., 2017; Stastnik et al., 2020; Krepkova et al., 2021).

### 7.3 Cosmetics

In recent years, natural antioxidants have gained attention because of their harmful effects of synthetic antioxidants on the human body (Singh and Agarwal, 2009). Elastase and collagenase affect the regeneration or degradation of the extracellular matrix of the skin dermis, resulting in loss of skin tone, wrinkle formation, and loss of elasticity (Chambers et al., 2017). Studies have shown that silymarin inhibits collagenase and elastase to a lesser extent (Nichols and Katiyar, 2010; Drouet et al., 2019).

### 7.4 Foods

The young leaves of *S. marianum* are tender, juicy, crisp, and refreshed, which are excellent vegetables for consume (Liu et al., 2016). Achenes of *S. marianum* are protein-rich and can be used to produce protein powder, which is characterized by high protein content, low fat content, and low cholesterol levels (Liu, 2021). According to Krepkova et al., combining oil of *S. marianum* with baking can increase the nutritional value of food by providing extra vitamins, proteins, and linoleic acid (Krepkova et al., 2021).

### 7.5 Others

**FIGURE 9:**
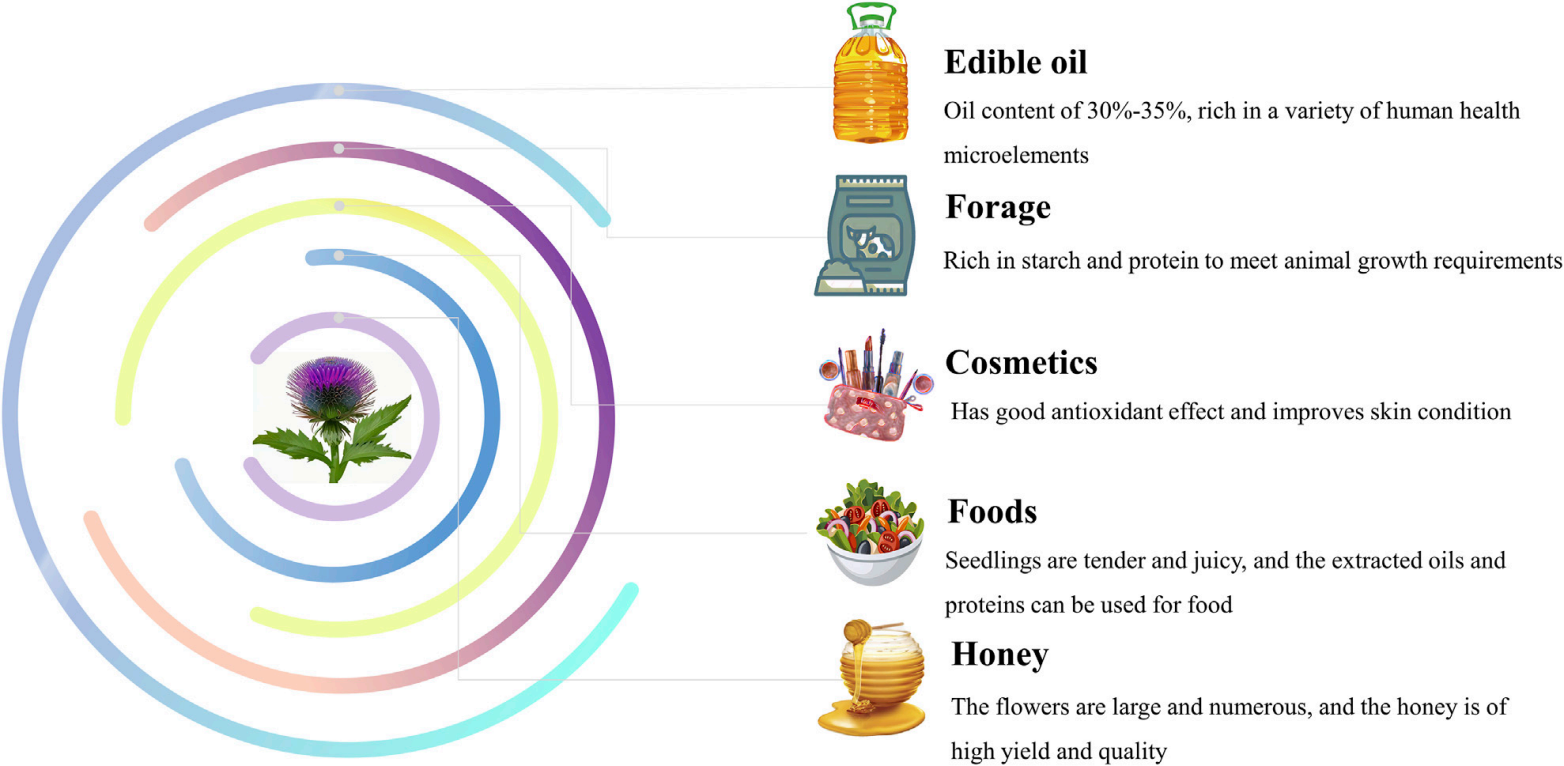
Comprehensive utilization of *S. marianum*.

*S. marianum* can reach heights of 1.5–2.0 m and quickly form barriers that are impassable to livestock within 2 months. The flowers are large, brightly colored, and numerous, with a long bloom period, making them suitable for ornamental purposes. Additionally, the plant can be used as green manure after oil extracted from the achenes, which provides the soil with a rich source of nutrients, promotes crop growth, and improves soil quality. *S. marianum* is a valuable honey plant owing to its high yield and potential health benefits, such as liver and stomach protection. Therefore, it is a promising source of nectar for the development of new honey products (Pereira et al., 2015). (Figure 9).

## 8 Conclusion and prospects

This research analyzed the status of *S. marianum* researches in recent years. More than 20 types of flavonolignans constituents have been isolated from *S. marianum*. *S. marianum* has been found to have a variety of pharmacological effects, including hepatoprotective, cardioprotective, anti-inflammatory, anticancer, antioxidant, immunomodulatory, and neuroprotective effects. In addition to playing a role in the field of medicine, *S. marianum* is also used to produce edible oil, protein powder, forage and so on. Its excellent antioxidant effect is also very suitable for making cosmetics to protect the skin.

In recent years, researchers have been committed to the development and utilization of *S*. *marianum*, but there are still many aspects that are not perfect. First, the quality of *S. marianum* is a hot issue of concern. It is necessary to adjust measures to local conditions to find the most suitable areas for plant growth, to ensure the yield and quality of medicinal materials, and to promote the healthy development of medicinal plants and regional economy. The second is that the planting technology is not perfect, and the mature cultivation experience of the original producing area can be used for reference to ensure the growth and survival of *S*. *marianum*. The third is the development and utilization of chemical components. At present, there are more than 20 types of flavonolignans isolated from *S*. *marianum* (Wang et al., 2020b). The discovery of these components provides more possibilities for the functional research of *S*. *marianum*. The extraction method of the active ingredient is continuously optimized to improve the extraction rate of silymarin-related components. Silymarin is commonly extracted by degreasing the achenes of the plant and extracting them using methanol (Wianowska and Wiśniewski, 2015). Higher yields and purity of silymarin can be obtained using the chemo-enzymatic method (Biedermann et al., 2014). Secondly, except for the main active ingredient silybin, the role and mechanism of other components are not clear enough. It is of great significance to clarify the role and mechanism of these components, and more efforts are needed in the future. Finally, the difference in the content of active ingredients in the achenes of *S*. *marianum* in different regions and the difference in the content of active ingredients in different varieties in the same region are also worthy of our research, which provides a theoretical basis for the production of higher quality medicinal materials and the breeding of excellent varieties of *S*. *marianum*.

In clinical medication, the therapeutic effect of *S. marianum* on the liver has been repeatedly verified in the long-term of medication practice, but the improvement of bioavailability still needs continuous research. Researchers are improving the bioavailability of silymarin through nanocrystals, nanosuspensions and solid dispersions, and complexes of cyclodextrins and phospholipids. In particular, the combination of silymarin with phosphatidylcholine increased the bioavailability of silymarin 4.6-fold compared to the extract alone (Javed et al., 2011; MacDonald-Ramos et al., 2021). Chemotherapy is a conventional means of cancer treatment, but there are also obvious drawbacks. Because of its strong toxicity, poor targeting, many side effects and difficult to control, the vast majority of patients develop drug resistance, which ultimately leads to the failure of chemotherapy. Therefore, plant-based therapeutic agents with low toxic and side effects, high anticancer activity and synergistic effect with anticancer drugs are the focus of our current research. In addition, other pharmacological effects of silymarin, such as prevention and treatment of diabetes, protection of myocardial cells, anti-platelet aggregation, anti-oxidation, and gastric protection, have not been widely valued and utilized. Therefore, researchers should conduct in-depth research on this and fully tap its medicinal value in order to better serve the majority of patients.

Due to the limited genetic information about *S*. *marianum*, the biosynthesis and regulation mechanism of silymarin has been difficult to elucidate. Exploring and revealing the genetic information in *S*. *marianum* is of great significance for the development of silymarin biosynthesis. In addition to medicinal use, *S. marianum* is also a multi-purpose economic crop, which has many application values such as ornamental, animal husbandry, food and healthcare. The plants of *S*. *marianum* are tall, gorgeous flowers, strong resistance, can be used for urban greening. The oil of *S*. *marianum* is rich in nutrients and a variety of beneficial ingredients, which can be used to produce edible oil, lubricating oil, soap and so on. *S*. *marianum* can also be used as nectar, feed, cosmetic raw materials, green manure and so on. The comprehensive utilization of *S*. *marianum* is aimed at exploring more value of resources, protecting human health and promoting regional economic development.

In summary, *S*. *marianum* is an important resource for human health. Its chemical compositions, pharmacological mechanisms, and biosynthesis need to be further studied in order to provide a theoretical basis for the development of medicinal functions of *S*. *marianum*. This review provides a valuable background for the research of *S*. *marianum*, and provides a reference for further research and application of this medicinal plant.

## Figures and Tables

**Table:** 

**CDK**	Cyclin-dependent kinases
**CTL**	Cytotoxic T lymphocyte
**DEN**	Diethylnitrosamine
**DOX**	Doxorubicin
**ER**	Estrogen Receptor
**ECM**	Extracellular matrix
**Fas**	Fatty Acid Synthase
**GSH**	Glutathione
**HCC**	Hepatocellular carcinoma cells
**HGF**	Hepatocyte growth factor
**IL**	Interleukin
**LDH**	Lactate dehydrogenase
**Lts**	Leukotrienes
**LOP**	Lipoperoxides
**MAPK**	Mitogen-activated protein kinase
**NK**	Natural killer
**PSA**	Prostate specific antigen
**Akt**	Protein kinase B
**ROS**	Reactive oxygen species
**ALDH1A1**	Aldehyde dehydrogenase 1 family, member A1
**TGF β**	Tubuloglomerular feedback
**TNF-α**	Tumor necrosis factor
**UVB**	Ultraviolet radiation b
**Caspase**	Cysteinyl aspartate specific proteinase
**CDKI**	Cyclin-dependent kinases inhibitors
**c-Met**	Cellular-mesenchymal epithelial transition factor
**Ets1**	Erythroblastosis-twenty six 1
**GPx4**	Glutathione peroxidase 4
**Her-2**	Human epidermal growthFactor receptor 2
**IF-1β**	Interferon beta-1
**IFN-γ**	Interferon gamma
**IGF-1**	Insulin-like growth factor 1
**IGFBP-3**	Insulin-like growth factor-binding protein 3
**JAK**	Janus kinase
**JNK**	C-Jun N-terminal kinase
**Ki-67**	Proliferation cell nuclear antigen
**TCF**	T-cell factor
**NF-κB**	Nuclear factor kappa B
**PI3K**	Phosphatidylinositol-3-kinase
**RARα**	Recombinant retinoic acid receptor alpha
**STAT3**	Signal transducer and activator of transcription 3
